# Clinical Features, Neuropsychology and Neuroimaging in Bipolar and Borderline Personality Disorder: A Systematic Review of Cross-Diagnostic Studies

**DOI:** 10.3389/fpsyt.2021.681876

**Published:** 2021-06-09

**Authors:** Anna Massó Rodriguez, Bridget Hogg, Itxaso Gardoki-Souto, Alicia Valiente-Gómez, Amira Trabsa, Dolores Mosquera, Aitana García-Estela, Francesc Colom, Victor Pérez, Frank Padberg, Ana Moreno-Alcázar, Benedikt Lorenz Amann

**Affiliations:** ^1^Institute of Neuropsychiatry and Addiction, Parc de Salut Mar, Barcelona, Spain; ^2^Centro Salud Mental Infanto-Juvenil, Parc de Salut Mar, Barcelona, Spain; ^3^Centre Fòrum Research Unit, Institute of Neuropsychiatry and Addiction, Parc de Salut Mar, Barcelona, Spain; ^4^Mental Health Research Group, Hospital del Mar Medical Research Institute (IMIM), Barcelona, Spain; ^5^PhD Progamme, Department of Psychiatry and Forensic Medicine, Universitat Autònoma de Barcelona, Barcelona, Spain; ^6^Centro de Investigación Biomédica en Red de Salud Mental, Madrid, Spain; ^7^Instituto de Investigación y Tratamiento del Trauma y los Trastornos de la Personalidad (INTRA-TP) Center, A Coruña, Spain; ^8^Departament of Basic, Evolutive and Education Psychology, Universitat Autònoma de Barcelona, Barcelona, Spain; ^9^Department of Psychiatry and Psychotherapy, Klinikum der Universität München, Munich, Germany; ^10^Department of Psychiatry and Forensic Medicine, Universitat Autònoma de Barcelona, Barcelona, Spain

**Keywords:** bipolar disorder, borderline personality disorder, clinical features, cognitive functions, neuroimaging, affective continuum

## Abstract

**Background:** Bipolar Disorder (BD) and Borderline Personality Disorder (BPD) have clinically been evolving as separate disorders, though there is still debate on the nosological valence of both conditions, their interaction in terms of co-morbidity or disorder spectrum and their distinct pathophysiology.

**Objective:** The objective of this review is to summarize evidence regarding clinical features, neuropsychological performance and neuroimaging findings from cross-diagnostic studies comparing BD and BPD, to further caracterize their complex interplay.

**Methods:** Using PubMed, PsycINFO and TripDataBase, we conducted a systematic literature search based on PRISMA guidelines of studies published from January 1980 to September 2019 which directly compared BD and BPD.

**Results:** A total of 28 studies comparing BD and BPD were included: 19 compared clinical features, 6 neuropsychological performance and three neuroimaging abnormalities. Depressive symptoms have an earlier onset in BPD than BD. BD patients present more mixed or manic symptoms, with BD-I differing from BPD in manic phases. BPD patients show more negative attitudes toward others and self, more conflictive interpersonal relationships, and more maladaptive regulation strategies in affective instability with separate pathways. Impulsivity seems more a trait in BPD rather than a state as in BD. Otherwise, BD and BPD overlap in depressive and anxious symptoms, dysphoria, various abnormal temperamental traits, suicidal ideation, and childhood trauma. Both disorders differ and share deficits in neuropsychological and neuroimaging findings.

**Conclusion:** Clinical data provide evidence of overlapping features in both disorders, with most of those shared symptoms being more persistent and intense in BPD. Thus, categorical classifications should be compared to dimensional approaches in transdiagnostic studies investigating BPD features in BD regarding their respective explanatory power for individual trajectories.

**Systematic Review Registration:** The search strategy was pre-registered in PROSPERO: CRD42018100268.

## Introduction

A recent meta-analysis of 42 studies (1) found that 21.6% of patients suffering from Bipolar Disorder (BD) fulfilled also diagnostic criteria for Borderline Personality Disorder (BPD), and conversely, 18.5% of BPD patients for BD. However, these data may not reflect true comorbidity given the overlap of their phenomenological features (2–4). Of note, researchers have also documented considerable rates of co-occurrence between BPD and Major Depressive Disorder (MDD) or BD ranging from as low as 3% to as high as 48% in clinical samples (5–8). The high frequency of diagnostic co-occurrence and resemblance of phenomenological features has led some authors to suggest that BPD is part of the bipolar spectrum (9–13). Other experts in the field have clearly opposed this notion (6, 14–17). Furthermore, in the latter line, recent narrative reviews concluded that both are distinct pathologies, even though they can be difficult to distinguish due to a considerable overlap between both conditions (3, 18). Differentiating BPD from BD-I appears relatively straightforward, reflecting the common presence of severe manic episodes, frequently with psychotic features, in BD-I. By contrast, BD-II (alternating depressive and hypomanic episodes) and BPD are frequently less precisely diagnosed, as BD-II patients do not present with psychotic symptoms and both share common clinical features, especially impulsivity and emotional dysregulation (19). Furthermore, in BD-II there is frequently subthreshold symptomatology between episodes instead of full remission. Therefore, misdiagnosis in both directions is common due to the uncertain boundaries between both disorders (13) and shared “transdiagnostic” features (3).

The main objective of this systematic review is to synthesize the evidence of whether clinical features, neuropsychological and neuroimaging data comparing both conditions point toward two distinct clinical entities or to them both belonging on a continuum within the affective spectrum.

## Materials and Methods

### Study Design

Using PubMed, PsycINFO and TripDataBase, we conducted a systematic literature search of studies published from January 1980 to September 2019 based on PRISMA guidelines (20) which compared clinical features, neuropsychological functions and neuroimaging between BD and BPD. The search terms were selected from the thesaurus of the National Library of Medicine (Medical Subject Heading Terms, MeSH) and the American Psychological Association (Psychological Index Terms) and included the terms “borderline personality disorder,” “BPD,” “bipolar disorder,” “BD,” “mania,” “hypomania,” “clinical features,” “clinical symptoms,” “emotional dysregulation,” “instability,” “temperament,” “mood,” “neuropsycho*,” “neurocognit*,” “cogniti*,” “impairment,” “deficit,” “functioning,” “cognitive function,” “executive function,” “attention,” “memory,” “working memory,” “neuroimaging,” “neuroimage.” The final search equation was defined using the Boolean connectors “AND” and “OR.”

The automatic search was later completed with a manual search of the reference list of previous reviews and meta-analysis. Titles, abstract, methods and results of the articles identified were screened for pertinent information. The search did not include any subheadings or tags (i.e., search fields “All fields”). In accordance with Cochrane Handbook guidelines, due to the significant heterogeneity and small number of the studies, a formal quantitative synthesis (i.e., meta-analysis review) was not possible to conduct (21), therefore a systematic literature review was conducted.

The Newcastle-Ottawa Scale for assessing the quality of non-randomized studies (22) was applied to each study separately by AMR and BH. Discrepancies were resolved by a third researcher, IGS. The completed PRISMA 2009 checklist (20) and the Newcastle-Ottawa Scale (22) are included in Supplementary Tables 1, 2.

### Inclusion Criteria and Exclusion Criteria

The selection of the articles was carried out using the following inclusion criteria: (i) observational studies published in peer-reviewed journals, (ii) human studies, (iii) adult populations (over 18 years), (iv) comparisons of a non-comorbid BD and non-comorbid BPD group in terms of (v) clinical features, (vi) neuropsychological functions or (vii) neuroimaging performance. The criteria for exclusion were: (i) articles that did not contain original research (i.e., reviews and meta-analyses), (ii) controlled studies, (iii) qualitative designs, (iv) empirical studies with quasi-experimental or single-case designs, (v) unpublished studies, (vi) in youth and child population, (vii) examined only pharmacological treatment trials. AMR and BH conducted the first literature search and selected the articles in an independent way. Discrepancies were resolved by IGS and AVG.

## Results

### Eligibility of Studies

**Figure 1:**
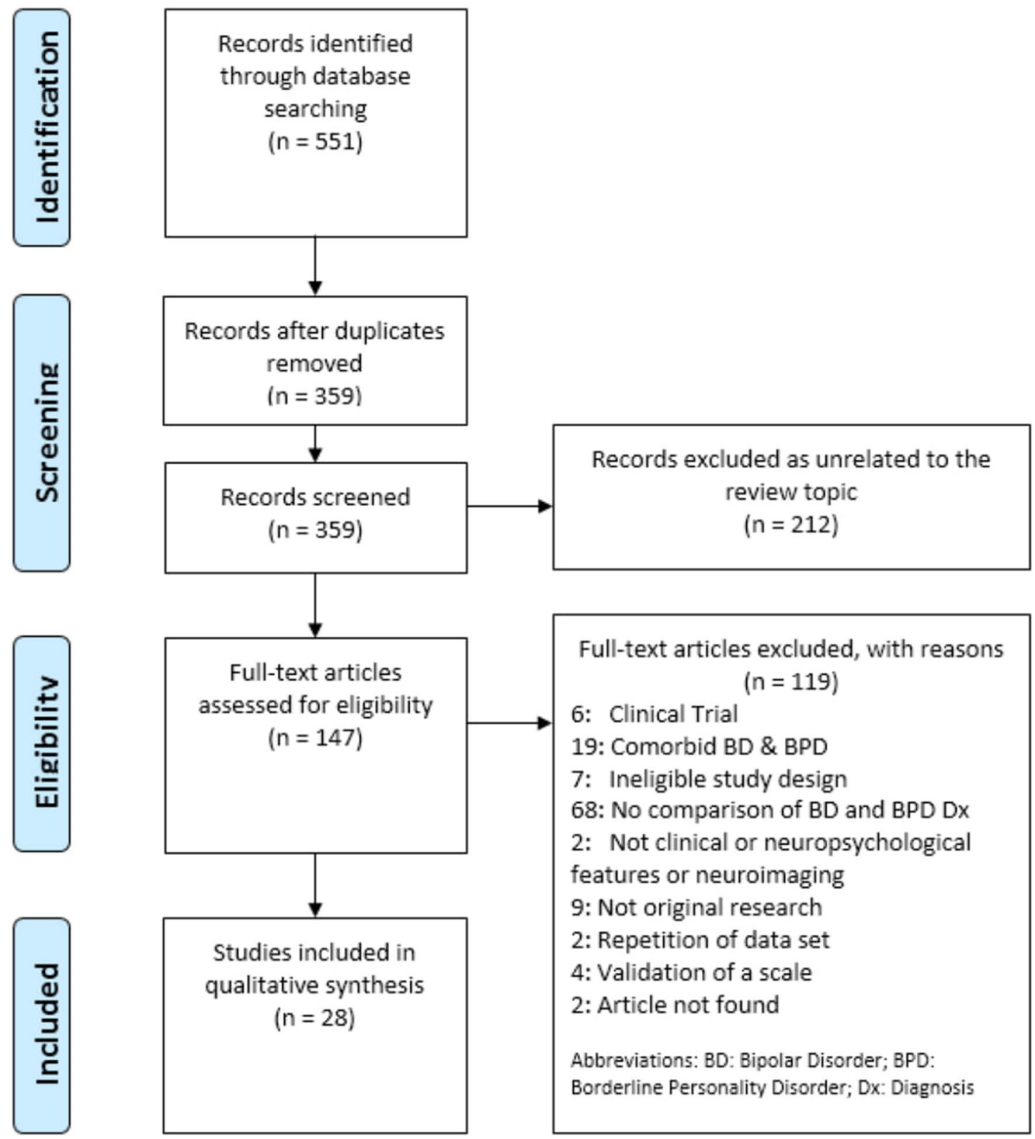
PRISMA flow diagram.

Using the search terms described above, the initial literature search produced 551 articles. Titles were screened for eligibility with the most relevant articles retained for abstract review. After an initial review of relevance, 359 articles were retained for further review of their titles and abstracts. BH, reviewer two, also screened articles independently from AMR. Discrepancies were resolved by two independent researchers, IG and AVG. 147 full-text articles were assessed for eligibility and a total of 28 studies published since 1980 met the inclusion and exclusion criteria: 19 focused on assessing clinical features (19, 23–39), six evaluated neuropsychological functioning (40–45) and three assessed similarities and differences in neuroimaging aspects (46–48). The study selection process can be seen in Figure 1.

### Clinical Features

Table 1 provides an overview of all studies mentioned in this section which evaluate clinical features in BD and BPD.

**Table 1:** Clinical features between bipolar disorder and borderline personality disorder.

**References**	**Sample**	**Variables**	**Assessment instruments**	**Main findings**
Bayes et al. (19)	DSM BD: 83 BPD: 53 CLIN EXTEND BD:125 BPD: 53 CLIN STRICT BD: 98 BPD: 53	• Mood symptoms	Structured Clinical Interview, Self-report	- BPD patients compared to BD were significantly more likely to report history of childhood sexual abuse, distant or rejecting parents and relationship difficulties. - BPD showed more suicide attempts, self-harming behavior and a younger onset of depressive symptoms.
Berrocal et al. (23)	BD: 16 BPD: 25 HC:39	• Mood spectrum	SCID-I, SCID-II, MOODS-SR	- BD and BPD patients had significantly higher scores for lifetime mood symptomatology than HC.
Pauselli et al. (24)	BD: 16 BPD: 16	• Clinical • Psychopathological features	SCID-II, BPRS, PANSS	- BD patients scored significantly higher than BPD on “euphoric manic” factor.
Vöhringer et al. (25)	BD: 118 BPD: 52	• Clinical features • Mood state	SCID-I, SCID-II, MDQ	- The elevated mood, increased goal-directed activities and periodicity are the strongest predictors in a mood setting for BD. - Racing thoughts, reduced need for sleep and an increased self-confidence were the weaker predictors of BD. - Female gender predicted BPD in a mood clinic setting.
di Giacomo et al. (26)	BD: 113 BPD: 248	• Depression • Anxiety • Mania	SCID-I, SCID-II, HAM-D, HAM-A, YMRS, BPDSI-IV	- BD patients in manic or mixed state scored in a very low range for depression and anxiety compared with BPD. - BD had low rates of impulsivity and anger control whereas higher rates of irritability and disruptive-aggressive behavior during acute manic phases. - BPD group showed greater affective instability and higher rate of suicidal and parasuicidal behaviors. Emptiness and identity disturbance as key symptoms.
Perroud et al. (27)	BD: 122 BPD: 116	• History of childhood maltreatment • Clinical history comorbid • Substance/Alcohol Use Disorder	SCID-I, SCID-II, CTQ	- BPD patients had more severe childhood maltreatment, younger age of onset of mood disorders and fewer overall mood episodes. - BD patients had a higher history of psychotic symptoms during a mood episode.
Saunders et al. (28)	BD: 20 BPD: 20 HC: 20	• Mood state • Clinical features • Cognitive ability • Cooperation	SCID-I, SCID-II, HAM-D, YMRS, BIS-11	- BPD in comparison with BD and HC scored lower on trait and state positive affect and higher on trait and state negative affect, higher impulsivity and aggression and reduced cooperative relationships. - BD patients scored higher than HC in trait negative affect, impulsivity and hostility and were more cooperative than HC. - BD and BPD had experienced significantly earlier physical or sexual abuse than HC.
Bachetti et al. (29)	BPD: 33 BD: 32	• Dysphoria	NDS-I	- Interpersonal Resentment dimensions of dysphoria were greater in BPD than BD.
Eich et al. (30)	BD-I: 17 BDII: 7 BPD: 27	• Temperament	TEMPS-A, SCID-I, SCID-II	- High cyclothymic temperament in BD and BPD. - BPD showed significantly lower levels of hyperthymia and higher levels of depressive temperament than BD. - BD had significantly lower anxious and irritable- explosive temperament than BPD.
Nilsson et al. (31)	BD-I: 25 BPD: 31 HC: 29	• Affective temperament • Maladaptive self- schemas	SCAN, SCID-II, TEMPS-A, YSQ-S3, BRManS, BRMeIS	- BPD showed significantly higher scores on cyclothymic, depression, irritable and anxious temperament than BD and HC. - BPD had lower scores on hyperthymic temperament than BD and HC. - BPD had higher scores on majority of maladaptive self- schemas. - BD showed higher scores than HC on cyclothymic temperament and insufficient self- control.
Mneimne et al. (32)	BD: 14 BPD: 38	• Emotional instability • Emotional reactivity • Emotional intertia	MINI, SIDP-IV, ESM	- With negative emotions, emotional instability has transdiagnostic patterns in BPD and BD. - BPD had heightened interpersonal reactivity to negative emotions (guilt, shame and excitement). - The emotional inertia of shame is a defining characteristic of BPD. - In BD dynamics of guilt and shame are not found.
Henry et al. (33)	BD-II: 13 BPD: 29	• Intensity shifts • Affective lability • Impulsivity • Aggressiveness	SIPD-R, SADS, ALS, AIM, BDHI, BIS- 7B	- BD-II was characterized by shifts from euthymia to depression and elation, and from elation to depression. - BPD was characterized by shifts from anger/anxiety to euthymia, presented significantly higher scores for affective intensity, impulsivity and hostility. - Significant interaction between the two disorders between anxiety and depression.
Reich et al. (34)	BDI-II: 24 BPD: 29	• Intensity shifts • Frecuency shifts	SCID-I, DIB-R, DIP-IV, AIM, ALI-BPD, ALS	- BPD showed significantly more frequent and intense affective shifts between euthymia-anger, anxiety-depression and depression- anxiety, and more intense shifts from euthymia to anxiety. - BD showed significantly more frequent and intense shifts from euthymia to elation.
Bayes et al. (35)	BD: 83 BPD: 53	• Emotion regulation • Cognitive emotion • Regulation	Semi-structured interview, MINI, DIP-IV, DERS, CERQ	- BPD displayed a higher number of maladaptive emotion regulation strategies. - Adaptive emotion regulation strategies were superior in BD group.
Fletcher et al. (36)	BD-II:24 BPD: 24	• Emotion • Perceived parental style • Depressive symptoms	MINI, DIPD-IV, CERQ, DERS, MOPS, QIDS-SR	- BPD significantly more likely than BD to use maladaptive emotion regulation strategies and less likely to use adaptive ones. - BPD patients scored significantly higher on majority of perceived parental style sub-scales than BD. - Dysfunctional maternal relationships related to maladaptive emotion regulation strategies in BPD. - Dysfunctional paternal relationships significantly associated with emotion regulation strategies in both clinical groups.
Kramer (37)	BD: 25 BPD: 25 HC: 25	• Coping processes	SCID-II, MINI, SCL-90-R, CAPRS	- BPD and BD had significantly lower coping functioning than HC in terms of competence and resources. - BPD patients had significantly lower coping functioning than BD patients in autonomy.
Bøen et al. (49)	BD-II: 20 BPD: 25 HC: 44	• Mood criteria • Impulsivity	MINI, SCID-II, MADRS, YMRS, CTQ, PDQ-4, NEQ, AUS, DUS, UPPS	- BPD patients showed higher levels of self-reported impulsivity than BD-II patients, who in turn showed more impulsivity than HC. - In BD-II group, increased impulsivity was strongly associated with depressive mood state and moderately with childhood trauma. - No association between impulsivity and childhood trauma in the BPD group.
Richard-Lepouriel et al. (38)	BD: 276 BPD: 168 HC: 47	• Impulsivity • Traumatic childhood experiences	SCID-II, DIGS, BIS-10, CTQ	- BPD and BD: impulsivity associated with adverse childhood experiences - Higher impulsivity in BPD than BD and HC - BPD: Impulsivity is an intrinsic trait - BD: impulsivity is environmentally driven and associated with adverse traumatic childhood experiences
Mazer et al. (39)	BPD: 20 BD: 16 HC: 15	• Emotional abuse • Physical abuse • Sexual abuse • Emotional neglect • Physical neglect • Symptomatology	SCID-II, BAI, BDI-II, BHS, BSI, YMS, CTQ	- BPD patients had a higher severity of depressive, suicidal ideation, hopelessness, anxiety and impulsivity symptoms than BD. - BPD experienced more severe early distress compared to BD patients. - A history of ELS in BPD and BD. - Higher proportion of ELS in BPD than BD and HC group.
				- Emotional abuse, emotional neglect and physical neglect predominated in BPD. - Physical and sexual abuse, only up to moderate severity, with no difference between clinical groups.

*ADHD, Attention Deficit Hyperactivity Disorder; AIM, Affective Intensity Measure; ALI-BPD, Affective Lability Interview for Borderline Personality Disorder; ALS, Affective Lability Scale; ASQ, Attachment Style Questionnaire; AUS, Alcohol Use Scale; BAI, Beck Anxiety Inventory; BD, bipolar disorder; BDHI, Buss-Durkee Hostility Inventory; BD-I, Bipolar Disorder Type-1; BD-II, Bipolar Disorder Type- 2; BDI-II, Beck Depression Inventory Second Edition; BHS, Hopelessness Scale; BIS-11, Barrat Impulsiveness Scale; BPD, Borderline Personality Disorder; BPDSI-IV, The Borderline Personality Disorder Severity Index-IV; BPRS, Brief Psychiatric Rating Scale; BRMaS, Beck Rafaelsen Mania Scale; BRMelS, Beck Rafaelsen Melancholia Scale; BSI, Suicidal Ideation Scale; CAPRS, Coping Action Patterns Rating Scale; CERQ, Cognitive Emotional Regulation Questionnaire; Clin extend, clinical extend; Clin strict, clinical strict; CTQ, Childhood Trauma Questionnaire; DERS, Difficulties in Emotion Regulation Scale; DIGS, Diagnostic Interview for Genetic Studies; DIPD-IV, Diagnostic Interview for Personality Disorders DSM-IV; DIS, Diagnostic Interview Schedule; DSQ, Defense Style Questionnaire; DSM, Diagnostic and Statistical Manual of Mental Disorders; DUS, Drug Use Scale; ELS, Early Life Stress; ESM, Experience Sampling Methodology; HAM-A, Hamilton Anxiety Rating Scale; HC, healthy control; HDRS, Hamilton Depression Rating Scale; IAS-R, Interpersonal Adjective Scales-Revised; MADRS, Montgomery- Åsberg Depression Scale; MDD, Major Depressive Disorder; MDQ, Mood Disorder Questionnaire; MINI, Mini-International Neuropsychiatry Interview; MOODS-SR, The Mood Spectrum Self-Report; MOPS, Measure of Parental Style; NDS-I, Neapen Dysphoria Scale- Italian Version; NNEQ, Network Entry Questionnaire; OPD, Other Personality Disorders; PANSS, Positive and Negative Syndrome Scale; PDQ-4, Personality Diagnostic Questionnaire Version 4; PIP, Physician Interview Program; QIDS- SR, Quick Inventory of Depressive Symptoms Self-Report; RIT, Rorschach Inkblot Test; SADS, Schedule for Affective Disorders and Schizophrenia; SASB, Structural Analysis of Social Behavior Rating Scales; SCAN, Schedules for Clinical Assessment in Neuropsychiatry; SCID-I, Semi-structured Clinical Interview for DSM Axis 1; SCID-II, Semi-structured Clinical Interview for DSM Axis II; SCL-90-R, Symptom Checklist 90; SIPD-R, Structured Interview Personality Disorders for DSM-III-R; SIPD-IV, Structured Interview Personality Disorders for DSM-IV; TCI, Temperament and Character Inventory; TEMPS-A, Temperament Evaluation of the Memphis; Pisa, Paris and San Diego Autoquestionnaire; UPPS, The UPPS Impulsive Behavior Scale; YMS, Young Mania Scale; YMRS, Young Mania Rating Scale; YSQ-S3, Young Schema Questionnaire-version 3*.

#### Affective Symptoms

Mood swings are prominent clinical features of both disorders, with affective symptoms of particular interest when comparing BPD and BD-II. In our review, eight clinical studies investigated general psychopathology comparing BPD and BD, in most cases without a healthy control group for comparison. Symptoms of mania and depression were evaluated mainly through clinical judgment, and other symptoms (e.g., dysphoria) through self-report.

Firstly, Berrocal et al. (23) found both disorders showed elevated lifetime mood symptomatology as compared to Healthy Controls (HC) but no significant difference between them.

Four studies compared both disorders in terms of manic symptoms (23–26). Berrocal et al. (23) found the differences in manic symptoms between bipolar patients and BPD were not statistically significant. Conversely, three of these found that these symptoms could differentiate the two disorders (24–26). Specifically, Pauselli et al. (24) found in an acute inpatient sample that manic-psychotic symptoms differentiated BD-I from BPD. Vöhringer et al. (25) in an outpatient sample, found that elevated mood, increased goal-directed activities, racing thoughts, reduced need for sleep, increased self-confidence and episodicity predicted BD but not BPD. Similarly, di Giacomo et al. (26) found that BD patients in mixed or manic phases showed significantly more manic symptoms than BPD patients.

At the other end of the mood spectrum, six studies compared the two disorders in terms of depressive symptoms (19, 23, 24, 26–28). Of these, two studies (23, 24) which did not state the BD phase, found no significant differences in depressive symptoms between the two disorders, including symptoms of inhibited depression or mixed features with anxiety, depression and suicidality. In contrast, di Giacomo et al. (26) found fewer anxiety and depressive symptoms in BD patients in manic or mixed states, while BPD patients had a wider range of anxiety and depressive symptoms. Similarly, two studies found BPD was characterized by a significantly earlier onset of depressive episodes than BD (19, 27), but BPD patients had fewer overall episodes and less history of psychotic symptoms during a mood episode. Furthermore, Saunders et al. (28) found that BPD patients had more depressive symptoms than euthymic BD patients and HC.

Finally, one study (29) compared the two disorders in terms of dysphoria. This study demonstrated that total score for dysphoria, and subscales of irritability and interpersonal resentment, were significantly higher in BPD patients than in BD.

Thus, in brief, manic features, including psychotic symptoms, can indicate a diagnosis of BD-I and distinguish BD from BPD. Otherwise, anxiety, depressive symptoms and dysphoria can be found in both disorders but may be more persistent, intensive and with an earlier onset of depressive symptoms in BPD than in BD.

#### Affective Temperament

The affective temperament is described as the set of behavioral traits, stable throughout the life course and with a biological basis, that reflect styles of affective reactivity including activity levels, mood rhythms and patterns of cognitive functioning (50–52). Assessed by the studies in this review by self-report measures, it has been discussed as a neurobiological underpinning of both BD and BPD (29).

Two studies compared the affective temperament of BPD with that of BD (30, 31). Eich and colleagues (30) suggested that BD and BPD share a common temperamental diathesis, including similar levels of abnormal cyclothymic temperament, reactive instability, anxious-dependent and avoidant attitudes, and impulsive reactive behavior. The other study (31) found that, while these disorders share abnormal temperamental traits, BPD patients showed a greater severity and scored significantly higher in terms of cyclothymic, depressive, irritable and anxious temperament than BD patients, whereas BD patients scored significantly higher in the hyperthymic temperament.

In conclusion, both conditions share high scores in various temperamental traits, but with a greater severity in cyclothymic, depressive, irritable and anxious temperament in patients with BPD, while the BD profile is characterized more by a hyperthymic temperament.

#### Emotion Dysregulation

Emotion dysregulation or affective instability is defined as the inability to flexibly respond and manage emotions (53) which interfere with appropriate goal-directed activity (54). Many researchers have characterized BPD and BD separately in terms of heightened reactivity and instability of negative emotion (55–58). In this review, seven studies reviewed affective instability/emotional dysregulation, with a set of structured diagnostic interview and self-rating scales, comparing BPD with BD patients (26, 32–37).

Although findings from four studies showed similarly high levels of affective lability comparing BPD to a mixed sample of BD-I and BD-II (26, 32–34), the results revealed also some different pattern in both disorders. Accordingly, Mneimne et al. (32) found that, while emotional instability is a shared trait, in terms of heightened instability of anger and irritability and heightened inertia of irritability, only BPD is characterized by emotional reactivity to interpersonal challenges, heightened for guilt, shame and excitement, irritability and happiness. As well, shifts from anger and anxiety to euthymia were associated with BPD, whereas shifts from euthymia to depression and elation, and shifts from depression to elation, were characteristic of BD patients (33, 34). Furthermore, BPD patients showed higher affective intensity (33, 34) and higher affective instability than BD patients in a mixed or manic state (26).

Two other studies analyzed similarities and differences between emotion regulation strategies in both disorders (35, 36). Both studies found that BPD subjects displayed a higher number of maladaptive emotion regulation strategies than BD patients, such as deficits in non-acceptance of emotional responses, limited access to emotion regulation strategies, lack of clarity around emotions and emotional awareness, difficulties in controlling impulsive behavior and a tendency to self-blame, catastrophize and blame others. BPD patients were also less likely to use adaptive strategies such as planning, reappraisal and putting things into perspective compared with those with a BD disorder. However, neither of these two studies compared patients to HC.

Coping, as a specific operationalization of affect regulation, was reviewed in one study (37). Findings showed that while both BPD and BD patients both had difficulties in the coping domains of competence and resources, BPD patients have a specific lack of adaptive autonomy coping patterns, such as negotiation and accommodation, as compared to BD patients.

In summary, the results show that emotional dysregulation or affective instability appear to be an overlapping symptom, but it is more pronounced in BPD and pathways are different. Furthermore, BPD have a higher number of maladaptive emotion regulation strategies than BD patients.

#### Impulsivity

Impulsivity or impulsiveness is a core feature of BPD and BD and may represent a way of managing negative emotions (9). It reflects the tendency to act on a whim, displaying behavior characterized by little or no forethought, reflection, or consideration of the consequences (59). Nine studies investigated impulsiveness in BPD and BD patients using clinical interviews and self-report measures (24, 26, 28, 31, 33, 35, 36, 38, 49).

Five of these studies directly compared impulsivity between BD and BPD and all of them found that BPD patients had elevated impulsivity compared to BD patients (24, 26, 33, 34, 36). Otherwise, four studies comparing BD and BPD patients with HC found that while BD patients had lower impulsivity than BPD, they showed significantly higher impulsivity than HC (28, 31, 38, 49). Further analysis of impulsivity profiles found that BPD patients demonstrated high levels of impulsivity as a trait, unrelated to current mood state, while impulsivity in the BD-II group is present as both a trait and strongly related to current depressive mood state (49). BPD patients had higher scores in all impulsivity dimensions except in novelty seeking (38). Both Bøen et al. (49) and Richard-Lepouriel et al. (38) found that impulsivity was correlated with levels of childhood trauma in BD, while in BPD it was not.

Similarly, three studies compared both disorders in terms of aggression, closely related to impulsivity (26, 28, 33). Two studies showed that BPD patients are more hostile and aggressive than euthymic BD patients and HC (28, 33). However, a further study found that BD patients showed low “anger-control” but high “irritability” and “disruptive-aggressive behavior” during manic or mixed phases (26).

Along these lines, two studies carried out into behaviors related to suicide and self-harm found that BPD patients show a significantly higher rate of self-injury, suicidal and parasuicidal behaviors than BD (19, 26).

In summary, there is strong evidence for a higher impulsiveness in BPD than in BD, which is again more pronounced when compared to HC. Whereas, in BPD high levels of impulsivity were described as a trait, in BD impulsiveness was potentially more related to affective episodes. Both disorders were related with aggressive behavior, which was again more intense in BPD.

#### Childhood Trauma

History of childhood trauma has been reported among the etiological factors of BD (60) and BPD (61). Five retrospective studies were carried out to investigate different forms of traumatic childhood experiences in both disorders using structured clinical interview and self-report questionnaires.

Five studies explored childhood trauma rates in BD and BPD patients (19, 27, 28, 39, 49). While both patient groups had experienced significantly more trauma than HC (28, 39), results point to severity of childhood trauma being more marked in BPD patients (19, 27, 28, 39, 49). In detail, Bayes, McClure et al. (19) found BPD patients were significantly more likely to report childhood sexual abuse, parental indifference, maternal abuse and over-control, developmental trauma, childhood depersonalization, distant/rejecting parenting. Similarly, Mazer et al. (39) found that emotional abuse, emotional neglect and physical neglect predominated in BPD, differentiating it from BD. Otherwise, Bøen et al. (49) found more severe childhood maltreatment in BPD. Conversely, Saunders et al. (28) found no significant difference between physical and sexual abuse suffered between both groups, although rates were higher in the BPD group.

Interestingly, Fletcher et al. (36) showed that maladaptive strategies and maladaptive emotion regulation were linked to parental style in both disorders, particularly in BPD, but with differences in terms of the type of trauma and its consequences. Therefore, dysfunctional maternal relationships characterize BPD, whereas dysfunctional paternal relationships led to emotion regulation deficits in both disorders. Furthermore, maternal abuse was associated with increased self-blame, while maternal over-control was associated with an increased tendency to catastrophize and blame others in BPD. Otherwise, paternal abuse was associated with a reduced use of acceptance strategies in BPD, while paternal over-control was negatively associated with the use of positive refocusing and to put things into perspective and a reduced impulsive control behavior in patients with BD-II. Finally, paternal relationships characterized by indifference were relevant to both groups, but in BPD this was associated with an increased tendency to blame others, whereas in BD-II it was associated with an increased tendency to ruminate.

Therefore, to summarize, both BD and BPD patients are more likely to have suffered childhood trauma than HC, but with BPD patients being more affected than BD.

#### Relationships With Self and Others

Three clinical studies compared BD and BPD in terms of different aspects of relationships with others (19, 28, 31) using interviews and self-report measures. Two studies found that BPD patients had significantly more difficulties in interpersonal relationships as compared to BD patients (19, 28). Specifically, Bayes, McClure et al. (19) found that two personality characteristics, “relationship difficulties” and “sensitivity to criticism by others,” distinguished BPD patients from BD patients and were the most consistent predictors of BPD. Saunders et al. (28) found that BPD patients showed reduced cooperation compared to BD patients and HC, and had difficulties in establishing and maintaining reciprocally cooperative relationships. In contrast, the results for euthymic BD patients showed they were capable of developing cooperative relationships and were marginally more cooperative than HC.

Additionally, Nilsson et al. (31) found that BPD patients endorse negative and distressing beliefs about themselves and their relationships with others compared to HC and BD group.

In summary, the evidence shows that BPD patients have more negative attitudes toward others and self, and more conflictive interpersonal relationships, which may distinguish BPD from BD.

### Neuropsychological Functions

Six studies investigated differences in executive functioning, attention and impulsivity between BD and BPD patients (40–45) and compared to HC data (Table 2).

**Table 2:** Neuropsychological differences between bipolar disorder and borderline personality disorder.

**References**	**Sample**	**Variables**	**Assessment instruments**	**Main findings**
Akbari et al. (40)	BPD: 35 BD II: 5 HC: 30	• Cognitive flexibility • Set-shifting • Response inhibition • Problem solving • Decision-making • Sustained attention • Selective attention	WCST, IGT, SCWT, ToL, CPT, WAIS.	- BPD and BD-II had poorer performance than HC on most neurocognitive domains. - BPD patients had more elevated response inhibition deficits and more impulsivity than BD-II patients. - BPD and BD-II patients had poorer performance in planning and problem-solving than HC.
Feliu-Soler et al. (41)	BD-I: 34 BD-II: 4 BPD: 35 HC: 70	• Neuropsychological performance	DIB-R, CPT-II	- BPD had reduced ability to discriminate stimuli and faster processing time. - BD showed significantly slower processing time. - Both clinical groups showed more omission, comission and perseveration errors than HC.
Gvirts et al. (42)	BD-I: 26 BD-II: 4 BPD: 32	• Neuropsychological performance • Functionality	CGI, GAF, CANTAB	- BPD showed deficits in planning compared to BD and HC and in problem-solving compared to norms of HC. - BD had significant deficits in strategy formation and increased execution time compared to BPD and norms of HC.
Lozano et al. (43)	BD: 19 BPD: 20 HC: 19	• Social, occupational and psychological functioning	GAF, HSCT, Flanker task	- BD and BPD showed significantly poorer interference control with more context-related errors than HC. - Interference score correlated with illness duration in BD.
Saunders et al. a (44)	BD I: 20 BPD: 20 HC: 20	• Clinical features • Neurocognitive performance	BIS- 11, Buss- Perry Q, Ravens' matrices, Reaction time task	- BPD patients showed transitory post-error slowing than BD and HC.
Saunders et al. (45)	BD: 20 BPD: 20 HC: 20	• Impulsivity • Decision-making	Raven's matrices, BIS-11, Buss- Perry Q, Risk choice task	- BPD were associated with problems attending to and using explicit reinforcement cues compared to BD or HC. - BPD had alterations in the processing of information about potential gains and losses compared to the other groups. - BD group was intermediate between HC and BPD in sensitivity to high-loss risks.

*BD, bipolar disorder; BD-I, Bipolar Disorder Type-1; BD-II, Bipolar Disorder Type-2; BIS-11, Barrat Impulsiveness Scale; BPD, Borderline Personality Disorder; CANTAB, Cambridge Neuropsychological Test Automated Battery; CGI, Clinical Global Impressions; CPT, Continuous Performance Test; CPT-II, Conner's Continuous Performance Test; DIB-R, Diagnostic Interview for Borderline-Revised; GAF, General Assessment of Functioning; HC, healthy control; HSCT, Hailing Sentence Completion Test; IGT, Iowa Gambling Task; SCWT, Stroop Color- Word Interference Test; ToL, Tower of London; WAIS, Weschler Adult Intelligence Scale; WCST, Wisconsin Card Sorting Test*.

Akbari et al. (40) found that euthymic patients with BD-II and BPD had a poorer performance than HC in most neurocognitive domains, specifically in cognitive flexibility and set-shifting, decision-making, sustained and selective attention and problem-solving. In addition, BPD patients had more elevated response inhibition deficits than BD-II patients, which may contribute to greater impulsivity and poor affect regulation.

Another study compared errors made on the Continuous Performance Test-II (CPT-II) related to impulsivity and attention in both disorders (41). The authors found both clinical groups displayed neuropsychological deficits as compared to HC, but that the pattern differed according to each disorder. Processing speed was a key differentiator, with BPD patients showing significantly faster processing speed which was related to impulsivity, and a reduced ability to discriminate stimuli, probably related to selective attention. On the other hand, BD patients showed greater overall cognitive impairment, with slower processing speeds and deficits in sustained attention. These results support the findings from studies into clinical features indicating impulsivity as a trait marker of BPD. BPD patients are characterized by high levels of motor impulsivity and non-planning impulsivity, while BD patients have increased levels of attentional impulsiveness (41).

Gvirts et al. (42) used the Cambridge Neuropsychological Test Automated Battery to compare sustained attention, problem-solving, planning, strategy formation, cognitive flexibility and working memory. Similar to the previous study, the authors found deficits in both disorders which followed divergent patterns. BPD patients showed greater deficits in planning compared to BD and HC, and in problem-solving only with the HC. Otherwise, BD patients showed significant deficits in strategy formation and increased execution time compared to BPD patients and HC. Both BPD and BD patients showed deficits in sustained attention as compared to HC.

Another study compared interference control between BPD, BD and HC assessing the ability to exert control over interference arisen from semantic memory or from distracting perceptual information (43). They found that BD and BPD shared a common impairment in the first task, but both disorders retained intact control over perceptual interference, leading the authors to conclude that similar cognitive functioning may underlie different disorders and symptomatology.

Saunders et al. (44) carried out a study to investigate the speed and accuracy of sensorimotor performance in BD patients, BPD patients and HC. They found no significant differences between any group in any task. However, BPD patients showed transitory post-error slowing.

Finally, Saunders et al. (45) carried out another study to evaluate decision-making in BPD patients, euthymic BD patients and HC. They found that BPD is associated with impaired decision-making, specifically in problems attending and using reinforcement cues to identify negative outcomes. They paid significantly less attention to prospective losses; thus BPD is related to engagement in harmful and risky behaviors. The BD sample showed no impairments in decision-making as compared to the HC participants, but were intermediate between the other groups in their sensitivity to high-loss risks. However, in this study the potential presence of comorbid Attention Deficit Hyperactivity Disorder (ADHD) is a possible confounder.

To conclude, evidence from the six neuropsychological studies shows that both disorders overlap and differ in neuropsychological deficits. BPD and BD-II patients had in common poorer performance in their planning and problem-solving performance, when compared to HC. Whilst deficits related to motor and non-planning impulsivity, planning, and difficulty attending to specific cues are more prevalent in BPD patients, deficits related to processing speed and strategy formation and increased levels of attentional impulsiveness are indicative of BD patients.

### Neuroimaging

Neuroimaging studies are increasingly used to characterize psychiatric disorders since they provide precise and direct information on the structure and functioning of the brain, which can be compared in different psychiatric disorders. Three studies revised neuroimaging features in BPD and BD as compared to HC (46–48) (Table 3).

**Table 3:** Neuroimaging results between bipolar disorder and borderline personality disorder.

**References**	**Sample**	**Variables**	**Method**	**Main findings**
Rossi et al. (46)	BD: 15 HC for BD: 15 BPD: 26 HC for BPD: 26	• Hippocampus volumetry	ROI analysis	- Smaller hippocampal volumes in both clinical groups than HC but with distinct patterns of gray matter loss (subiculum and right dentate gyrus).
Rossi et al. (47)	BD: 14 HC for BD: 14 BPD: 26 HC for BPD: 26	• Brain volumetry	VBM RV analysis	VBM - BD vs. HC: Smaller GM in temporal and frontal lobes, precuneus, cerebellum and thalamus. - BPD vs. HC: Smaller GM in hippocampus, amygdala, prefrontal, parietal and occipital lobes. RV - BD, BPD vs. HC: Smaller global GM - BD vs. BPD: less GM volume in the frontal, parietal, limbic and cerebellar regions.
Das et al. (48)	BD: 16 BPD: 14 HC: 13	• Functional connectivity	Resting state	- BD and BPD were differentiated on the basis of resting state functional connectivity among networks. - BD had an increased connectivity in networks related to social understanding, but diminished emotional clarity. - BPD displayed decreased connectivity in networks responsible for self-referencing information and failure to integrate information.

*BD, bipolar disorder; BPD, Borderline Personality Disorder; HC, Healthy Control; ROI, Region of Interest; VBM, Voxel-Based-Morphometry; RV, Regional volumes*.

Rossi et al. (46) carried out a structural Magnetic Resonance Imaging (MRI) study to explore volumetric differences in hippocampal subdivisions in BPD and BD as compared with HC using a three-dimensional mapping method. Results showed that both clinical groups had smaller hippocampal volumes as compared to HC; however, two distinct patterns of gray matter (GM) loss were found in each of the clinical conditions. More specifically, the hippocampal surface maps showed that the CA1 region and the subiculum were bilaterally atrophic in BPD, whereas in bipolar subjects there was a significant alteration in the right dentate gyrus.

In order to extend the previous analysis, the same research team carried out another structural study using a Voxel-Based-Morphometry (VBM) and Regional Volumes (RV) analysis (47). The VBM analysis showed again distinct patterns of GM alterations for each condition. BD subjects presented smaller GM volume in the temporal and frontal lobes, and the precuneus, cerebellum and thalami regions compared to the HC. Otherwise, BPD subjects showed smaller GM volume in the hippocampus, the amygdala and the prefrontal, frontal, parietal and occipital lobes. However, the RV analysis showed that both disorders presented smaller global GM regional volumes compared with HC. The results also provided evidence that regions selectively observed in BPD are strongly correlated with deficits in emotional processing (hippocampus, middle and inferior temporal gyrus). Furthermore, the RV analysis showed that BD had less GM volume in the frontal, limbic, parietal and cerebellar regions compared with BPD. The authors hypothesized that BD might present a relatively diminished pre-frontal modulation of subcortical and medial temporal structures within the limbic system (amygdala and thalamus), resulting in dysregulation of mood.

Likewise, Das et al. (48) carried out a study in order to understand how impaired functional connectivity correlated with emotion dysregulation in both disorders. Results demonstrated that BD and BPD can be differentiated on the basis of resting state functional connectivity among networks involved in detecting social salience, self-referential processing and emotional regulation. Specifically, BD patients displayed increased connectivity, compared to both BPD and HC, in coupling of social salience (SS)-ventral medial prefrontal cortex (vmPFC) and default mode (DM)-precuneus networks. Conversely, BPD patients displayed decreased connectivity as compared to the rest of the groups in the coupling of SS-precuneus and SS-right fronto-parietal (RFP) networks that were responsible for self-referencing information and which were related to the failure to integrate information from the environmental stimuli and internal representation, linked to impulsive behaviors and self-harm.

In conclusion, both structural and functional neuroimaging findings suggest that a number of areas of overlap exist; however, structural and functional findings also suggest neuroanatomical differences between BPD and BD which could explain the clinical symptomatology presented in both disorders, especially related with emotional processing and emotional dysregulation. Nevertheless, the sample size for significance of these studies are limited, meaning a firm confirmation about neuroanatomic differences still cannot be drawn.

A summary of the specific and shared features of both disorders can be seen in Table 4.

**Table 4:** Specific and shared features of bipolar disorder and borderline personality disorder.

**Feature**	**Bipolar disorder**	**Borderline personality disorder**	**Bipolar disorder and borderline personality disorder**
Manic-psychotic symptoms	BD- I: Manic-psychotic symptoms: - elevated mood - increased goal- directed activities - racing thoughts - reduced need for sleep - increased self-confidence - mood episodicity	Less history of psychotic symptoms during a mood episode.	
Affective symptoms	BD-I: a wider range of anxiety and depressive symptoms.	- An earlier onset of depressive episodes than BD. - Fewer overall episodes than BD. - Higher rates of dysphoria, irritability and interpersonal resentment.	Mixed features with anxiety, depression and suicidality common to both disorders
Temperament	Higher hyperthymic temperament.	Greater severity of cyclothymic, depressive, irritable and anxious temperament than BD.	Common temperamental diathesis - abnormal cyclothymic temperament - reactive instability - anxious- dependent and avoidant attitudes - impulsive reactive behavior
Emotional instability	- At least a 4-day mood course - Shifts from euthymia to depression/ elation - Shifts from depression to elation - Triggers of emotional instability not clear	- Mood tends to change over hours - Shifts from anger and anxiety to euthymia - Emotional reactivity to interpersonal challenges	Emotional instability is a shared feature but expressed differently in each disorder
Impulsivity	- Impulsivity is related to current mood state - Related to disinhibition	- Higher levels of impulsivity, as a trait - More hostile and agressive - Higher rate of self-injury, suicidal and parasuicidal behavior - Tends to decrease over time	Impulsivity a shared feature with a different expresión in each disorder
Coping strategies	Higher number of adaptive strategies than BPD: - planning - reappraisal - puting things into perspective. - ruminate	Higher number of maladaptive emotion regulation strategies than BD: - non-acceptance of emotional reponses - limited access to emotion regulation strategies - lack of clarity around emotions and emotional awareness - difficulties in controlling impulsive behavior - a tendency to self-blame catastrophize and blame others.	Shared difficulty in coping but difficulties are in different areas
Childhood trauma	Higher rates of childhood trauma than in controls	More likely to report: - childhood sexual abuse - parental indifference - maternal abuse and over-control - developmental trauma - childhood despersonalization - distant/rejecting parenting	A shared feature, more marked in BPD
Neurocognitive deficits	- Slow processing speed - Deficits in sustained attention - High levels of attentional impulsiveness. - Significant deficits in strategy formation. - Sensitivity to high loss risks	More elevated response inhibition deficits than BD-II. - Fast processing speed - Reduced ability to discriminate stimuly - High levels of motor impulsivity and non-planning impulsivity. - Deficits in planning - Problems attending to and using reinforcement cues to identify negative outcomes	BD-II and BPD have shared difficulties in these neurocognitive domains: - cognitive flexibility and set-shifting - decision- making - sustained and selective attention - problem- solving - impairment in exerting control over interferences arising from semantic memory - Evaluating decisión making
Neuroimaging data	- Significant alteration in the right dentate gyrus. - Small volume in the temporal and frontal lobes, precuneus, cerebellum and thalami regions - increased connectivity in coupling of social salience-ventral medial prefrontal cortex and default mode precuneus networks	- CA1 region and the subiculum were bilaterally atrophic. - Small GM volume in the hippocampus, the amygdala and the prefrontal, frontal, parietal and occipital lobes. -Lower connectivity in the coupling of social salience-right fronto-parietal networks	Smaller hipocampal volumes than healthy controls and functional connectivity deficits

## Discussion

To our knowledge, this is the first systematic review of clinical and neurobiological studies which directly compare patients with BPD and BD. A total of 28 studies met our inclusion criteria, 19 of them comparing clinical features, six comparing neuropsychological performance and three comparing neuroimaging abnormalities. Our review shows that both disorders can be distinguished in terms of distinct clinical variables, but also share a variety of psychiatric symptoms. Interestingly, most of the shared symptoms seem more persistent and intense in BPD. Furthermore, while neurobiological data suggest differences and similarities between both disorders, the data from the neuropsychological and especially neuroimaging studies are currently limited. This means we cannot definitively answer our research question of whether both conditions belong to the same affective continuum, or whether they are separate nosological entities, with BD a mood disorder and BPD a personality disorder.

Most clinical variables investigated were present and common in both disorders, but there exists a clear tendency that various symptoms are more intense in BPD and some variables differ in further phenomenological and state/trait aspects. For instance, BD patients present more mixed or manic symptoms than BPD patients. A manic state, related to euphoric mood with psychotic symptoms, increased goal-directed activities and mood episodicity, seems specific to distinguish BD-I from BPD (3, 24, 25). Of note here, affective instability in BPD shifts from anger and anxiety to euthymia, whereas in BD it shifts from euthymia to depression and elation, and from depression to elation (33, 34, 62). What is more, the temporal course of emotional dysregulation differs between the two disorder: in BPD mood tends to change over hours, usually in response to interpersonal conflict, while in BD it requires at least a 4-day course to be described as hypomania and the triggers are less clear (63). This distinct pattern might represent a relevant underpinning for the differentiation between BD-I and BPD. Interestingly, patients with BPD show more maladaptive regulation strategies in their affective instability than BD patients. Unlike BPD, impulsivity is not a diagnostic criterion of BD, but it could be related to disinhibition, present during manic or mixed episodes (64, 65) and also persistent during euthymia (66) yielding problematic behaviors in both disorders such as self-injury, suicidality or substance abuse (66, 67). Results showed differences in profile impulsivity between both disorders, and in this regard our neuropsychological findings are of note. As stated, BPD patients are characterized by high levels of motor impulsivity and non-planning impulsivity while BD patients have increased levels of attentional impulsiveness (41). This finding could be related with cognitive execution. Along these lines, BPD patients are characterized by a faster processing speed and a reduced ability to discriminate stimuli and to use cues to identify negative outcomes, while in BD there are more deficits in sustained attention. Therefore, impulsivity can be considered more as a trait in BPD, while in BD it can be considered more as a state and related to affective episodes. Interestingly, impulsivity in BPD tends to decrease over time while affective characteristics and relationship problems are more likely to continue, unlike BD. Further evidence which appears to support two different disorders might be that BPD patients have an emotionally noxious sense of self, more negative attitudes toward others and self, and more conflictive interpersonal relationships than bipolar patients. There is an extensive literature that has attempted to clarify the nosological controversy between bipolar disorder and borderline personality disorder. While older articles support the vision of a mood spectrum (9–13), others, including more recent ones, tend toward the idea that they are two different entities (6, 14–17). Our findings to this point represent a tendency so far toward a more dichotomous approach to both disorders, especially to BD-I and BPD. Putting our findings into the context of reviews which included data from studies which investigated the disorders separately rather than directly comparing them, our findings that mood lability, emotional dysregulation and impulsivity are symptoms common to both disorders (although their presentation and time course may be different), but that there are differences in terms of manic and psychotic symptoms and past abuse, are in line with recent reviews (3, 18). Again using data from studies that were not a direct comparison, previous authors have also found differences in parasuicidal self-harm, incidence of suicide, and treatment response, and more data to support a difference in cognitive deficits, as well as differences in bipolar family history suggesting a stronger genetic component in BD (3, 16). Indeed, the heritability rate in bipolar disorder has been shown to be greater than in BPD (between 0.69 and 0.80 (68) compared to between 0.35 and 0.80 (69, 70). Finally, a recent narrative review by Sanches (18) also summarizes that the two conditions are separate, but also states: “It is possible that some forms of bipolar disorder are virtually identical to BPD from a phenotypical standpoint, making both conditions difficult to distinguish at times, particularly given the absence of well-established biomarkers from both conditions.”

In this sense, we detected various clinical variables which are prevalent and common in both disorders and differ in intensity only. This observation could serve as an argument to place both conditions along a continuum of the affective spectrum, as proposed by Akiskal (9), or following Sanches (18) who suggested that some forms of BD are identical to BPD from a phenotypical standpoint. Clinical variables common to both are aggressive behavior and suicidal ideation. Furthermore, symptoms in both disorders include depressive and anxious symptoms, dysphoria, and hypomanic mixed states. The latter are defined by high and low symptoms concurrently present in the same episode (10, 71). Whilst we have evidence to be able to differentiate BPD from BD-I, the difference between BPD and BD-II is much more challenging and often confusing in clinical settings. Especially in cases of a pronounced affective instability or rapid cycling (four or more affective episodes a year), both conditions present with the aforementioned affective symptoms. An important neurobiological underpinning might hereby be the underlying abnormal temperamental traits, which are considered as genetically stable and are hypothesized as etiological risk factors for both BPD and BD (29). We found evidence that BD-II and BPD share higher scores in all affective temperamental traits, with a greater severity in cyclothymic, depressive, irritable and anxious temperaments in patients with BPD and, conversely, the hyperthymic temperament being more specific to BD. Akiskal (9) hypothesized hereby that BPD belongs to the affective spectrum as “the dysphoric facet of cyclothymia” due to the extreme emotional reactivity in BPD, which is based on abnormal affective temperament and cyclothymia (with brief alternating hypomanic and depressive symptoms) in both disorders.

Childhood trauma, such as emotional abuse and neglect, is associated with both BPD and BD and, specifically, with emotional regulation difficulties and propensity to impulsiveness (38, 72, 73). While both patient groups had significantly experienced trauma, results point to the severity of abuse being more marked in BPD patients. In our review, preliminary neuroimaging evidence shows that BPD and BD share overlapping functional and structural neuroanatomical abnormalities, especially involving temporal lobe and related limbic structures associated with mood lability and rapid cycling. These abnormalities could be associated with childhood maltreatment and, in turn, with high sensitivity to rejection, and with impulsiveness, all characteristic factors of both BPD (38, 66, 72, 73) and BD (49, 61, 66, 73, 74). Based on these results, impulsivity could be conceptualized as a consequence of malfunctioning emotion regulation mechanisms or even as a facet of emotional dysregulation, rather than an expression of impulsivity as a primary trait. However, results diverge here as well. Plasma cortisol levels and sexual abuse were correlated in both diagnostic-related groups, but in opposite ways. In BPD, the level of cortisol was positively correlated with sexual abuse, while in BD the correlation was negative. Thus, in BPD patients the history of sexual abuse could stimulate the functioning of the HPA axis while in BD patients a more inhibitory response is observed. Additionally, a negative correlation was identified between emotional neglect and physical neglect, and plasma cortisol levels in BPD patients.

These results highlight the need to research further the role of childhood trauma in the nosology of these disorders and are in line with the previous data on the etiology of these disorders. Recent reviews suggest that BD could be the result of a *gene x environment interaction*, supporting the evidence from this review that environmental factors such as childhood trauma and adverse life events play a significant role (75–77), while a recent review by Cattane et al. (61) suggests that BPD is a result of a combination of biological vulnerabilities and environmental factors including the exposure to traumatic experiences during childhood. Early life stress and childhood trauma have been seen to be major risk factors for the development and persistence of BPD, contributing to the emergence of hippocampal abnormalities. In this sense, the subiculum modulates the response to stress, influencing the hypothalamic-pituitary-adrenal (HPA) axis and being the primary locus of hippocampal interaction with this axis (61). On the other hand, atrophy in the dentate gyrus is associated with deficits in the GABAergic system which have been implicated in the etiology of BD (46). Additionally, the impairment in the former network was related to deficits in conscious self-representation and in turn with impaired social understanding, while impairment in the second network explained emotional clarity deficits and depressive rumination (48). In this line, these results could explain why bipolar patients have heightened awareness and increased sensitivity and receptivity to social inputs, but because of diminished emotional clarity, they are unable to process the meaning of these inputs with respect to internal emotional milieu. Nevertheless, few studies have been carried out in this direction, so the results must be interpreted with caution and are not robust enough to draw firm conclusions. Greater understanding of the etiology of the two disorders in the future may help answer our research question, especially in light of the fact that debate over the clinical similarities and differences has gone on for a long time without a decisive consensus being reached.

Other variables should be also mentioned here, even though they have not been targeted in this review. One is longitudinal data. A review by Parker (17) regarding whether BPD can be classified as a mood disorder argues that longitudinal data helps clarify the distinction between BPD and BD-II. On the other hand, a recent article suggests the course of BPD frequently changes over time, with symptoms fluctuating to such an extent that they no longer fulfill the diagnostic criteria later in the life course (78), unlike BD which tends to be a lifelong disorder. As Diagnostic and Statistical Manual of Mental Disorders (DSM) criteria state that traits in personality disorders are chronic and pervasive (63), this puts into doubts the nature of BPD as a personality disorder, and could be considered as a further argument for summarizing BPD within a broad affective spectrum with a shift from an Axis II to Axis I disorder. In our review, all the studies which met the inclusion criteria were cross-sectional, and it would be interesting to see longitudinal research directly comparing the two disorders.

Furthermore, pharmacological treatment and psychotherapy in BPD is worth mentioning due to the nosological nature of our review and its frequent use in clinical practice. Ghaemi et al. (3) argue certain treatment effects may be diagnostically specific. They support there being a strong consensus that psychotherapies alone are not effective in BD but they may be effective adjunctively with medications while, in contrast, psychotherapy is central to the treatment for BPD and there exists controversy about the benefits of psychopharmacotherapy (3). As well, a recent review of pharmacological treatment and psychotherapy in BPD recommends in general terms the use of both strategies (79). The authors highlight the need for further large randomized controlled pharmacological trials across classes of medication (especially in antidepressants) in BPD, but state that the available evidence suggests that anticonvulsants (mainly valproate and lamotrigine), atypical antipsychotics (especially olanzapine) are therapeutic options in treating impulsivity, anger, and affective instability in BPD patients. All these agents are indicated and frequently used in BD. This is of interest as core symptoms of both BPD and BD disorders can improve with medication (80).

However, the different psychotherapeutic treatment options appear to favor a more dichotomous approach to both conditions. The disorders differ in terms of the triggers of mood change. In BPD, most of the mood swings are associated with interpersonal triggers, while in BD they are related more often to biological factors linked to sleeping habits, self-care and changes of season. Thus, BD psychotherapy would address risk factors and environmental triggers to reduce emotional dysregulation (81) while BPD psychotherapy would be focused on social coping and trouble resolution strategies in the form of Dialectic Behavior Therapy (DBT) to improve emotional regulation (82). This would again support the idea that in BPD, the mood tends to change over time and in response to interpersonal conflict, whereas in BD the change lasts for a few days and the triggers are less clear. On the other hand, and based on the similarities observed in cognitive performance in both conditions, some studies have included cognitive rehabilitation and cognitive remediation techniques in their intervention. A systematic review carried out by Bellani et al. (83) states that there is evidence of functional and cognitive improvements in patients with BD who have undergone cognitive remediation treatment. Pascual et al. (84) carried out a controlled study of cognitive rehabilitation in patients with BPD, observing also an improvement in the psychosocial functioning of these patients. These results have led the authors to propose the possible efficacy of broader interventions which might help both disorders and include the elements of the psychoeducational model, DBT emotional regulation techniques and, in turn, cognitive remediation strategies.

Strengths of this work include that this is, to the best of our knowledge, the first systematic review that involves only studies which directly compare both disorders and review clinical and neuropsychological features and neuroimaging data. Due to the comparative lack of data of these last two categories, most of the discussion focuses on the clinical features. This review comprises a total sample of 2,631 participants which provides interesting preliminary evidence and directions for future research. Another strength of the review was the search of three databases for articles over an extended timeframe, repeated by an independent reviewer with a third to resolve discrepancies.

Limitations of our work need to be taken into account and include a lack of longitudinal studies and that several studies had no HC group, meaning an estimation of the degree of impairment or clinical severity of the patients is difficult. Several studies did not confirm the diagnosis of one or both clinical groups or confirm the lack of psychopathology in the control group. Additionally, studies differed in how the BD group was defined, with the majority not distinguishing between BD-I and BD-II in the statistical analysis, while others focused on BD-II or cyclothymia, making generalizations difficult, as well as differences in whether BD samples were euthymic or in an acute phase. Many studies did not compare BD and BPD during depressive episodes, when there are many overlapping symptoms (85). Three studies did not confirm diagnosis as part of the study (24, 26, 37), while four studies did not screen the control group for psychopathology (23, 31, 46, 47). There could have been comorbidity or a BD/BPD diagnosis in these groups which would have confounded results. A large variety of diagnostic tools and measures and scales were employed between the studies. Some studies relied on self-report measures for some or all outcome variables. All childhood maltreatment data was retrospective and self-report, meaning they may be subject to recall bias and an individual's subjective interpretation of events (86), which may have limited validity (87). Pharmacological treatment, the first-line treatment for BD and often used to stabilize mood in BPD (71), may have confounded results and was controlled for only in a minority of studies. Additionally, some studies used only female samples, meaning findings may not be generalizable to males. As well, two studies (19, 35) appeared to use the same sample, as did the group of Saunders et al. (44, 45) and the group of Rossi et al. (46, 47), meaning findings may be distorted. Finally, only three imaging studies are included in the review. One study (48) was performed with fMRI at resting state in a sample of 16 BD patients and 14 BPD, and two studies by almost the same authors (46, 47) were performed with sMRI in the same small sample of patients and controls. The sample size for significance of both functional and structural studies was not reached, meaning these studies were underpowered, thus the conclusions of these studies cannot be generalized to the general population of BD and BDP patients.

## Conclusion

In conclusion, based on current evidence directly comparing the two disorders, there is not sufficient data to either confirm the distinction as separate nosological entities, or to support both disorders being part of a shared affective spectrum continuum, or BPD being a bipolar sub-type. Although the results obtained in this exhaustive review may lead us to support the view of BD and BPD as two different categories, in clinical practice the differences between both are often very subtle, especially between BD-II and BPD. leading us to contemplate a dimensional vision.

Common criteria such as emotional dysregulation are easily identified but focusing on differential symptoms is a more complex task. BD-I differs clearly from BPD in the sense of manic/mixed phases which require hospitalization and affective instability has different pathways, being especially common interpersonal triggers in BPD. Furthermore, impulsivity seems more a trait in BPD, whereas a state in BD, and decreases over time in BPD, unlike in BD, supported by some neuropsychological findings. On the other hand, most symptoms, especially depressive, anxious, and hypomanic mixed symptoms are shared in both conditions (with a greater emphasis on BD-II than BD-I), even though they may be more intense in BPD. The latter might indicate that BPD is on the upper end of the affective spectrum as a temporal (as diagnosis of BPD changes over time), but more severe condition, whereas BD tends to be a lifelong disorder. Central shared parts of both conditions are affective temperament, even though some are more specific for one or the other entity, and adverse experiences in childhood, which have an especially important etiological role that is objectified in structural and functional brain alterations, which can explain symptomatic deficits. The noxious sense of self and difficulties in interpersonal relationships are characteristic of BPD. Neuropsychological, but especially neuroimaging, data are inconclusive so far to decide on the nosological discussion between both disorders. However, a recent Genome Wide Association Study by Witt and colleagues (88) revealed that “rather than being a discrete entity,” BPD has an etiological overlap with major psychosis, including BD. We must not forget other factors that can help us in the distinction as the family history of BD, the incidence of suicide and suicide attempts, the pharmacology response, the form of mood cycling or psychotic episodes or the incidence of early sexual abuse.

Future lines of investigation should focus on clinical and neuobiological variables with larger samples and hetero-applied scales, translating this information into effective diagnostic tests and treatment techniques for both disorders. It would be necessary to carry out more clinical trials focused mainly on neuropsychological and neuroimaging aspects directly comparing BD and BPD to increase the understanding of common and differentiated aspects between these two clinical conditions. In particular, prospective, longitudinal cross-diagnostic studies are needed to clarify the controversy. Some authors advocate for a multivariate approach that goes beyond the categorical distinction and uses dimensional information as a better way to understand the relationship between BPD and BD-II (89). Current diagnostic systems demonstrate a move toward dimensional rather than categorical approaches to classifying personality pathology and specially to consider personality traits in the management of all patients with BD irrespective of whether criteria for a categorical BPD are met (90). In this line, the alternative model proposed by the 5th edition of Diagnostic and Statistical Manual of Mental Disorders (63) and the 11th edition of International Classification of Diseases for Mortality and Morbidity Statistics (91) point to dimensions in BPD. Thus, another approach would investigate personality disorder dimensions in the BD spectrum.

## Data Availability Statement

The original contributions presented in the study are included in the article/Supplementary Material, further inquiries can be directed to the corresponding author/s.

## Author Contributions

AM and BH separately carried out the search and the primary assessment of the quality of non-randomized studies and wrote the first draft of the manuscript, supervised by AM-A. Two researchers, IG-S and AV-G, resolved discrepancies. AM-A, BA, VP, FC, FP, and DM, experts within the investigated field and contributed with the interpretation of data. AG-E and AT participated with contributions to the manuscript. All authors contributed to the article and approved the submitted version.

## Conflict of Interest

FP is a member of the European Scientific Advisory Board of Brainsway Inc., Jerusalem, Israel, and has received speaker's honoraria from Mag & More GmbH and the neuroCare Group. His lab has received support with equipment from neuroConn, Ilmenau, Germany, and Mag & More GmbH and Brainsway Inc., Jerusalem, Israel. The remaining authors declare that the research was conducted in the absence of any commercial or financial relationships that could be construed as a potential conflict of interest.
