# Supplementary material for: Clinical Features, Neuropsychology and Neuroimaging in Bipolar and Borderline Personality Disorder: A Systematic Review of Cross-Diagnostic Studies
Source: Front Psychiatry. 2021 Jun 9;12:681876. doi: 10.3389/fpsyt.2021.681876 (PMC8220090; doi:10.3389/fpsyt.2021.681876)
Supplement: Supplementary file 2 [file Table_2.docx]

**Supplementary table 2.** Newcastle-Ottawa Scale Quality Assessment Results.

|  | **Selection: Representativeness of the sample** | **Selection: Sample size** | **Selection: Non-respondents** | **Selection: Ascertainment of the exposure** | **Selection: Comparability and control of confounders** | **Selection: Assessment of the outcome** | **Selection: Statistical test** | **Total** |
| --- | --- | --- | --- | --- | --- | --- | --- | --- |
| Bayes, McClure et al., 2016 (19) |  |  |  | * | ** | * | * | ***** |
| Berrocal et al., 2008 (23) | * |  |  | ** | * | * | * | ****** |
| Pauselli et al., 2015 (24) | * |  |  |  | ** | ** | * | ****** |
| Vöhringer et al., 2016 (25) | * | * | * | ** | ** | * | * | ********* |
| di Giacomo et al., 2017 (26) | * | * |  | ** |  | * | * | ****** |
| Perroud et al., 2016 (27) | * |  |  | ** | ** | * | * | ******* |
| Saunders et al., 2015 (28) |  |  |  | ** | ** | ** | * | ******* |
| Bachetti et al., 2019 (29) | * |  |  | ** |  | * | * | ***** |
| Eich et al., 2014 (30) | * |  | * | ** | * | * |  | ****** |
| Nilsson et al., 2010 (31) | * |  |  | ** | ** | * | * | ******* |
| Mneimne et al., 2017 (32) |  |  |  | ** |  | * | * | **** |
| Henry et al., 2001 (33) |  | * |  | ** | ** | * | * | ******* |
| Reich et al., 2012 (34) |  |  |  | ** | ** | * | * | ****** |
| Bayes, Parker et al., 2016 (35) |  |  |  | ** | ** | * | * | ****** |
| Fletcher et al., 2014 (36) | * | * |  | ** | ** | * | * | ******** |
| Kramer, 2014 (37) | * |  |  | ** | ** | * | * | ******* |
| Richard-Lepouriel et al., 2019 (38) | * |  |  | ** | ** | * | * | ******* |
| Mazer et al., 2019 (39) |  |  |  | ** |  | * | * | **** |
| Akbari et al., 2019 (40) | * |  |  | ** | * | * | * | ******* |
| Feliu-Soler et al., 2013 (41) | * | * |  | ** | ** | ** | * | ********* |
| Gvirts et al., 2015 (42) | * |  |  | ** | ** | ** | * | ******** |
| Lozano et al., 2016 (43) | * |  |  | ** | ** | ** | * | ******** |
| Saunders et al., 2016a (44) |  |  |  | ** | ** | ** | * | ******* |
| Saunders et al., 2016b (45) |  |  |  | ** | ** | ** | * | ******* |
| Rossi et al., 2012 (46) | * |  |  | ** | ** | ** | * | ******** |
| Rossi et al., 2013 (47) | * |  |  | ** | ** | ** | * | ******** |
| Das et al., 2014 (48) |  |  |  | ** | ** | ** | * | ******* |
| Bøen et al., 2015 (49) | * | * |  | ** | ** | * | * | ******** |
